# Complexity of the Nano-Bio Interface and the Tortuous Path of Metal Oxides in Biological Systems

**DOI:** 10.3390/antiox10040547

**Published:** 2021-04-01

**Authors:** Joseph S. Erlichman, James C. Leiter

**Affiliations:** 1 Department of Biology, St. Lawrence University, Canton, NY 13617, USA; 2 White River Junction VA Medical Center, White River Junction, VT 05009, USA; james.c.leiter@dartmouth.edu

**Keywords:** cell trafficking, endocytosis, exocytosis, protein corona, redox chemistry

## Abstract

Metal oxide nanoparticles (NPs) have received a great deal of attention as potential theranostic agents. Despite extensive work on a wide variety of metal oxide NPs, few chemically active metal oxide NPs have received Food and Drug Administration (FDA) clearance. The clinical translation of metal oxide NP activity, which often looks so promising in preclinical studies, has not progressed as rapidly as one might expect. The lack of FDA approval for metal oxide NPs appears to be a consequence of the complex transformation of NP chemistry as any given NP passes through multiple extra- and intracellular environments and interacts with a variety of proteins and transport processes that may degrade or transform the chemical properties of the metal oxide NP. Moreover, the translational models frequently used to study these materials do not represent the final therapeutic environment well, and studies in reduced preparations have, all too frequently, predicted fundamentally different physico-chemical properties from the biological activity observed in intact organisms. Understanding the evolving pharmacology of metal oxide NPs as they interact with biological systems is critical to establish translational test systems that effectively predict future theranostic activity.

## 1. Introduction

There is tremendous interest in metal oxide nanoparticles (NPs) for use in therapeutic applications such as diagnostic tools and drugs in which the nanoparticles are either the active agent or passive, drug delivery nanocarriers. To-date, there are over thirty different metal oxide formulations being studied that may have biological effects [1], but few have garnered FDA clearance. While nanomaterials have demonstrated potential therapeutic benefit in many biomedical applications, clinical translation of individual formulation has not progressed as rapidly as one would expect given the plethora of preclinical studies [2,3]. We believe that the slow progression to approved drugs may result, in part, from the types of translational models used to study these materials, and the emerging evidence that the activity of nanomaterials in cell-free conditions and reduced preparations can be fundamentally different from the biological properties of the nanomaterial when studied in either cell culture conditions or, more importantly, in intact organisms. A better understanding of the evolving pharmacology of metal oxide nanoparticles (NPs) as they interact with biological systems is critical to establish translational test systems that can effectively predict future drug potential.

This review focusses on the impact of dynamic changes in chemical and physical properties of metal oxide NPs as they are distributed or transported from the point of administration to their intended targets. The ‘promise’ of metal oxides as potential nano-theranostics arises from their ability to participate in controlled, durable, biologically important redox reactions [4,5]. Often the chemical reactivity of metallic nanoparticles is presented as a binary function, either as oxidizing agents or as reducing agents, but the duality of the redox activity of metallic ions is often dictated by the conditions in which the material is studied, which may or may not reflect the biological environments relevant to the targeted pharmacological activity of the nanomaterial. In multicellular systems, this may involve successive passage through multiple cell membranes, extracellular barriers, intracellular organelles, and varied redox environments. As metallic NPs traverse biological barriers to their intended destinations, each nanoparticle acquires a protein coat, the composition of which changes subtly as the particle moves from the site of administration to the site of action. The dynamic nature of the protein corona can profoundly alter the biological properties of each NP including surface reactivity, particle aggregation, and cellular uptake, localization, retention, and toxicity. Not surprisingly, the activity of the material at the biological destination may be quite (and sometimes disappointingly) different from the biological activity present at the site of departure where the metallic NP entered the body. Thus, the most significant obstacle in the development of engineered nanomaterials as drug compounds is controlling how these materials interact with biomolecules. In this review, we provide a systematic review of the sources of biological variation introduced as nanoparticles traverse the tortuous pathway from the site of administration to site of action. Our purpose is ultimately to bring a more organized and rational approach to understanding why so many nanoparticles have begun with a bang but ended in a whimper.

## 2. The Origin of Biological Activity in the Structure of Nanoparticles

All nanoparticles, regardless of elemental composition or shape, have extremely high surface area:volume ratios that confer chemical reactivity not observed in particles with larger dimensions (i.e., > 100 nm) [6]. The solubility of nanomaterials in biological fluids is dictated by surface composition, surface charge, and the hydrophobicity/hydrophilicity profile. The surface electrostatic interactions between particles determine their propensity to aggregate and adsorb proteins to their surface. The chemical and biological reactivity as well as biodistribution of the nanomaterials are derived from these fundamental properties.

### 2.1. Redox Reactivity of Metal Oxides

Metal oxides NPs have an ability to participate in myriad biologically important redox reactions and mimic a wide range of enzymes including catalases, oxidases, dismutases, peroxidases, ATPases and phosphatases [4,7,8,9]. The native enzymes expressing similar redox activity play manifold and crucial roles in redox-dependent signaling cascades, and metal oxide NPs can disrupt or restore redox balance in cells through these reactions and signaling processes [9].

Not all metal oxides exhibit the same enzymatic activities, and mimetic activities can be ‘biased’ by the local environment surrounding the particle. There are three key factors that determine the interaction between the biological milieu and the redox activity at the nano-bio interface: the half-cell potential of the elements comprising the particle, the organization of the surface atoms of the nanoparticle, and the oxidation state of the ions on and within the nanoparticle. Reduction in the oxidation number of the metal (i.e., the accounting of the number of electrons a metal possess or lacks) occurs when the crystal loses an oxygen atom and forms a vacancy in the NP. Thermodynamically, any oxide is potentially reducible [10], and the distinction between reducible and non-reducible metal oxides depends on the ease with which oxygen vacancies can be formed [10]. In non-reducible metal oxides, the thermodynamic cost of formation of oxygen vacancies is high, and redox activity is absent [11]. In reducible metal oxides, oxygen vacancy formation is thermodynamically more favorable and occurs at lattice surfaces and edges where the coordination number of the surface atoms (i.e., the total number of bonds to the atom) is less than inside the crystalline structure of the oxide. The edge is also where lattice strain is highest [12]; all of which facilitates the formation of oxygen vacancies [10,13]. Thus, the highest enzyme-mimetic activity occurs at the surface of the nanoparticle [10,14]. Many transition-metal oxides, such as TiO_2_, MnOx, NiO, Fe_2_O_3_, and CeO_2_, are reducible because the energetic barriers to oxygen vacancy formation are low, and vacancies can occur spontaneously across the surface of the crystal.

The chemical mechanisms underlying redox activity can be divided into either electrophilic or nucleophilic reactions. Extra-facial, adsorbed oxygen is responsible for most of the electrophilic reactions, whereas interfacial oxygen, where lattice oxygen vacancies are created, underlie the nucleophilic reactions [15]. In general, nucleophilic oxygen (e.g., the oxide ion) is capable of carrying out selective oxidations while it seems that electrophilic oxygen species, which are deficient in electrons (e.g., the superoxide radical), appear to be more promiscuous and are largely responsible for non-selective oxidation [16]. There is usually a ‘preferred’ or stable oxidation state in each NP, and surface defects created by spontaneous loss of oxygen result in different valence states (i.e., Ce^4+^ > Ce^3+^). The redox state of the metal oxide can flip-flop repeatedly between valance states, which provide durable, recycling, catalytic activity.

The mechanisms of cyclic, regenerative redox reactions have been studied in cerium oxide NPs because of the relatively low barrier for the transition between Ce^4+^ 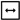 Ce^3+^. Cerium oxide demonstrates both superoxide dismutase and catalase mimetic activity [17,18,19]. In the reaction scheme shown below, the hydroxyl radical is the ‘seed’ for the balanced set of redox processes. Given the high oxidation potential of ^•^OH (+2.31 V at pH 7.0) and a high rate constant, hydroxyl radical can react rapidly with many biomolecules and lead to oxidative damage. However, the superoxide anion is likely the reactant with ceria. Superoxide anion is continually produced as a result of aerobic respiration, and the production of this reactive oxygen species, which functions as an important biological signaling molecule, can be greatly up-regulated in disease states. Superoxide dismutase converts superoxide anion into peroxide, and this species is quickly converted to water and oxygen by catalase or by reacting with hydroxyl anions via Fenton reactions. Both of these potent oxidizing agents, O_2−_ and H_2_O_2_, likely contribute to oxidative stress and damage of DNA, proteins, and lipids [20].

Formation of oxygen vacancies within the ceria nanoparticle lattice structure is central to this regenerative mimetic activity. The sequence of proposed reactions to explain the mimetic activity of cerium oxide is shown below [21,22]:

H_2_O_2_ + O_2_ +2OH^−^ → 2O_2_^−^ + 2H_2_O (1)

Ce^4+^ + O_2_^−^ → Ce^3+^ O_2_ (2)

Ce^3+^ O_2_^−^ + 2H^+^ → Ce^4+^ + H_2_O_2_ (3)

Cerium oxide acts as a catalyst, and so the sum of these reactions is:

H_2_O_2_ + 2O_2−_ + 2H^+^ → 2H_2_O + 2O_2_ (4)

Note that the ceria-dependent reaction start at an oxygen vacancy in the nanoparticle lattice; the hydroxyl ion represents the cellular source of oxidative stress; the superoxide radical reacts with both Ce^4+^ and Ce^3+^ in the dismutation process; hydrogen peroxide formed by superoxide dismutation reacts with Ce^3+^; both superoxide and hydrogen peroxide are consumed; the reaction is pH dependent; last, as pointed out by Reed et al. [22], the system of reactions is self-limiting (in the absence of a source of oxidizing agents like hydroxyl, there is little redox activity) and self-balancing. Other metal oxide NPs may have other preferred reactants so that the concentrations of a variety of oxidants, but especially the superoxide anion, may be reduced or regulated through multiple enzyme mimetic processes occurring simultaneously, even within the same NP.

**Figure 1:**
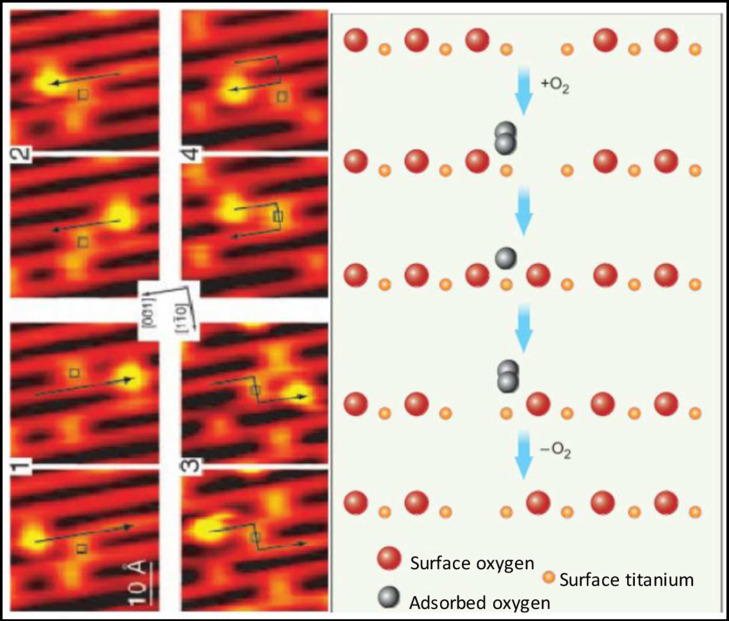
Scanning tunneling microscopic (STM) images (**left**) and molecular schematics (**right**) demonstrate the interactions of molecular oxygen adsorbed to the surface of titanium oxide at the site of oxygen vacancies within the crystal structure of the metal oxide. Each adjacent STM image shows the surface structure of the metal oxide before and after the interaction with molecular oxygen and the migration of the oxygen vacancy. Used with permission from Pinto et al. [10].

The oxygen vacancies in metal oxide NPs are transient and mobile across the surface of the metal oxide crystal, and the oxygen vacancies occur predominantly at the surface lattice boundaries or ‘edges,’ especially in smaller particles (~5 nm). The vacancies may then migrate internally where the coordination number with the metallic ions may be increased (Figure 1). The ability of the metal oxide to undergo reduction (vacancy formation), and the subsequent reincorporation of oxygen into the crystalline structure allows cyclically regenerative redox reactions, the durability of these reactions in vivo depends on particle retention in tissue and maintenance of the crystalline structure; dissolution of the crystal terminates redox reactivity.

In addition to reducing concentrations of oxidizing agents, most metal oxides can elicit free radical-mediated toxicity via the formation of hydroxyl through Fenton-type reactions [23,24]. Within reactive sites generated at oxygen vacancies, electron donor or acceptor regions interact with molecular O_2_ to form O_2_^•^—which in turn can generate additional reactive oxygen species (ROS). Fe_3_O_4_ magnetic nanoparticles, for example, exhibited intrinsic peroxidase-like activity under acidic conditions and a catalase-like activity at neutral pH [25]. Moreover, both hematite nano-Fe_2_O_3_ and maghemitenano-Fe_2_O_3_ induced hydroxyl radical formation in more acidic environments through Fenton reactions. The specific reaction that predominates (i.e., oxidation or reduction) will depend on the valence state of the crystal, which is modified by the pH of the cellular compartment in which the particle resides (as shown above for cerium oxide). Redox cell damage may also occur if dissociation of the metal ions (i.e., Ag NPs and Quantum Dots) elicits cellular enzyme deactivation, membrane disruption, altered electron transfer, reduced mitochondrial membrane potentials, or changes in gene expression; all of which may increase the accumulation of cellular oxidants [26,27,28,29,30,31,32].

The potential benefits of metal oxide nanoparticles for medical applications have emerged from their robust antioxidant properties [33,34,35]. Most studies fail to parse the impact of the local environment on nanoparticle reactivity and concentrate on the net effect of the nanoparticle as either pro- or anti-oxidant. This creates the (mistaken) impression that the metal oxide exhibits only one type of redox reactivity when in reality metal oxide NPs may have flexible redox reactivity that can be biased toward oxidation or reduction depending on the valence state and the milieu of the nanoparticle (pH, protein corona, cell-free media, serum, cell culture media, etc.).

### 2.2. Intracellular pH Environments and Metal Oxide NP Activity

**Figure 2:**
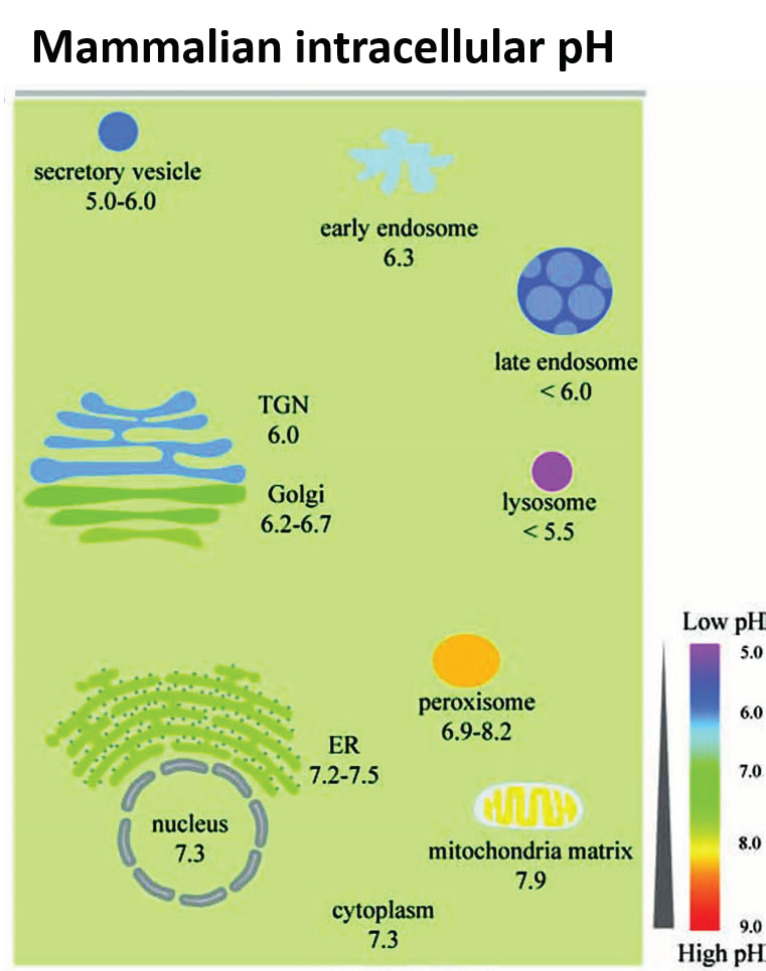
The range of pH values in intracellular compartments is shown schematically. The extent and type of chemical activity of metal oxide NPs may vary significantly, even in a single cell, across a wide range of pH values in different organelles. ER, endoplasmic reticulum and TGN, *trans*-Golgi network. From Shen et al. [41] with permission.

Fan et al. [33] synthesized Pt-Ft nanoparticles using an apoferritin protein shell/scaffold as a nanoreactor to control the synthesis of size-tunable Pt nanostructures. One to two nm Pt–Ft NPs synthesized in this way possessed both catalase and peroxidase activities. However, these superparamagnetic iron particles (SPIONs) demonstrated peroxidase activity in acidic solutions, but lost this activity in more neutral solutions and instead expressed catalase-like activity through a series of coupled reactions [33]. The antioxidant properties of CeOx NPs dominate at physiological pH, whereas these particles exhibit high oxidase activity at acidic pH [36], likely related to a net shift in the valence of material to Ce^4+^ [37]. Moreover, SOD activity is enhanced at lower pH relative to catalase activity, resulting in the accumulation of peroxide [38]. In more neutral conditions, CeOx NPs display both SOD and catalase activity [17]. Silver NPs were similarly sensitive to pH: the level of hydroxyl radical formation through a Fenton-like mechanism was dependent on pH-hydroxyl radical formation occurred at pH 4.6 or lower, but at more neutral pH, no significant formation of hydroxyl radicals occurred [39]. Thus, the tuning or biasing of the enzymatic mimesis of metal oxide NPs is modulated by intracellular pH, which can vary both by cellular localization (i.e., cytosol versus lysosome; see Figure 2) or whether the cells are immortalized or not [33,40].

### 2.3. Model System Effects

The biological effects of nanoparticles depend not just on the properties of the material in standardized conditions, but also on the biological system in which the nanoparticles are active [42,43,44,45,46,47]. There is increasing evidence that immortalized cells (i.e., differentiated cancer cells) have unique redox profiles that are different from their native, healthy counterparts [48,49]. Selective cytoprotection has been reported following administration of nanoceria in normal, healthy cells, but not in cancer cells [50,51]. Often, cancer cells rely more on glycolysis for energy production, and consequently they maintain more acidic intracellular pH values [52]. Where additional protons are present (i.e., lactate accumulation or localization in acidic organelles), Ce^3+^ reacts with a H^+^ and O_2_^•−^ to produce Ce^4+^ and H_2_O_2_, leading to net oxidation [38,53]. Moreover, in a comparison of immortalized colorectal cells (HCT 116) and human embryonic kidney (HEK 293) cells, CeOx NPs increased the ROS load and subsequently induced apoptosis in colon cancer cells but not in the embryonic kidney cells, suggesting that differences in either cellular localization or baseline pH existed in these cell types [54]. The accumulation of CeOx NPs in this study was not evaluated, so it is possible that the amount of material taken up by these two cell types could have differed and impacted ROS formation. In a study of three different MnOx NPs (MnO_2_, Mn_3_O_2_, Mn_3_O_4_) with different valance states, the biological implications of valence switching were examined in a cell-free system. Each MnOx NP exhibited both pro- and anti-oxidant activities simultaneously, including oxidase-, catalase-, and superoxide dismutase (SOD)-like activities. These MnOx NPs decreased cell viability in a dose-dependent manner in colorectal adenocarcinoma cells (Caco-2) regardless of valence, and the largest reduction in viability was associated with Mn_3_O_4_ > Mn_3_O_2_ > MnO_2_. While the MnOx NPs were all cytotoxic, they protected cells when the cells were challenged with peroxide—suggesting that catalase mimetic activity was protective [55]. Unlike many other metal oxides, the MnOx NPs were devoid of peroxidase or hydroxyl radical scavenging activity in cell-free assays, but when studied in cells, the MnOx NPs were located in the cytosol, which has a higher pH than most other organelles in the cell, and the local pH may have biased the enzyme mimetic activities of the different valences and allowed the particles to provide cytoprotective activity when the cells were challenged with peroxide. Consistent with these findings, MnOx nanoparticles increased catalase and SOD activities, while they also decreased glutathione levels in cell culture [56]. The decreases in cell viability caused by MnOx NPs were associated with mitochondrial dysfunction and apoptosis, presumably secondary to the reduction in glutathione levels. Glutathione is critical to maintain mitochondrial function and cell viability, and loss of sufficient glutathione levels in mitochondria increased oxidative stress [57]. Most often, MnOx NPs are cytotoxic in immortalized cell cultures, but the outcome of administration of these materials in whole animals is variable, and some studies show that they are safe (Xiao et al., 2013) but not others [58]. Hence, these nanoparticles may be protective in certain redox states and certain cell types but not others.

The variable redox effects of metal oxide NPs, which may be either pro-or antioxidant, have been vexing. Beyond the effects of the cells studied and the impact of pH in these test systems, redox activity of NPs may be related to the manner of synthesis (valence ratio), the size of the particles, the complement of adsorbed proteins, and the cellular localization of the material. The redox activity of metal oxide NPs is not easily predicted since local environments may vary so much. Moreover, findings in cell-free systems are not fully recapitulated in more representative biological environments like cell culture or intact animals. The biological impact of these materials seems to be tied to the baseline redox status of the cells being studied, which adds yet another source of variability when trying to characterize the likely therapeutic effect of nanoparticles. While many disease states elevate oxidative stress in tissues, not all tissues will have the same redox changes driven by the disease state. Thus, even within a single organism, the redox activity of a nanoparticle may differ organ by organ or even organelle by organelle. Since the delivery of metal oxides occurs passively, these materials distribute widely throughout the body including healthy cells, and healthy cells may be negatively impacted by NPs while the benefit of these materials as antioxidants may be observed only in cells that have a high oxidative load [48,49]. Understanding how these factors modify redox reactivity will be critical to the future development of therapeutic nanoparticles [59,60].

## 3. Biological Consequences of the Evolution of the Protein Corona

The transformation of a particle’s initial, synthetic reactivity to biological activity after interactions with proteins in a biological environment was brought to the forefront by Walkey et al. [61]. Blood plasma contains ~3700 different proteins [62,63], which are present at concentrations up to 60–80 g/l [64]. There are ample opportunities for a wide variety of protein-nanoparticle interactions, and the consequences of these interactions are far reaching. First, surface proteins may either increase the nanoparticle solubility or decrease it, thereby altering bioavailability. Second, they may impact cellular or system functions (i.e., immune system) and alter a myriad of cellular signaling cascades [65]. Third, protein-nanoparticle interactions may alter the chemical reactivity of the nanoparticle relative to the reaction profiles reported in cell-free systems [59,60]. The importance of the biological consequences of protein adsorption cannot be overstated, and the interactions of metal oxide NPs with proteins are central in understanding chemical and biological activity at the bio-nano interface. Finally, the biological identity of each nanoparticle is dynamic and evolves as the nanoparticle encounters different proteins in diverse cellular environments en route to its biological targets. Each biological environment that a particle enters and leaves generates a lasting protein “fingerprint” reflective of the path of the nanoparticle to its destination. The serum protein fingerprint depends primarily on the size and composition of the particle, and specific fingerprints may exist for each type of NP [61,66]. The route of administration (intravenous, sub-cutaneous, oral,) will also affect the composition of the proteins adsorbed to the particle.

The biological responses to a nanoparticle following assimilation are largely defined by the dynamic nature of the protein corona around the nanoparticle and the cellular uptake process that identify the protein-NP complex [67,68]. Approximately 95% of nanomaterials that are delivered to the blood are bound by elements of the complement cascade, are rapidly sequestered by reticuloendothelial organs (i.e., spleen and liver), and never reach their intended targets [69,70,71], unless the liver or spleen is the target. Given the passive tissue targeting of many nanomaterials, there can be many off-target organs and tissues as nanoparticles move toward the intended site of biological activity. As a consequence of protein-NP interactions, the chemical reactivity and enzyme mimetic activity of metal oxide NPs can be more easily assessed in cell-free systems. However, translating the chemistry of the NP in cell-free conditions into predictable behavior in less reduced biological systems becomes much more complicated where protein-NP interactions are unavoidable.

To varying degrees, all biological fluids contain proteins that will adsorb to the surface of nanoparticles, a process that is affected both by protein concentration, protein composition, and the nature of the solute. The suite of proteins adsorbed to the surface of the material changes dynamically over time as the NP is exposed to different proteins and solutes [72]. The adsorption kinetics of proteins and NPs have two phases: a rapid phase that occurs on the order of seconds to minutes and a slower phase that occurs over hours to days [73,74,75], and the protein corona around each NP has two layers, proteins associated with the ‘hard’ corona close to the nanoparticle are tightly bound to the particle surface whereas the ‘soft,’ outer protein corona is associated with protein-protein interactions at the interface of the hard corona and proteins in solution. Thus, the protein composition of the corona provides an evolutionary history of protein interactions and a fingerprint of the solutions through which the nanoparticle has passed [76].

The biological consequences of protein deposition are profound. The nanomaterial can change the conformation of the protein, thereby modifying the biological activity of the protein (i.e., Mac receptor activation fibrinogen [77]) or conversely, alter the surface chemistry of the nanoparticle either increasing or decreasing redox reactivity, affecting clearance from biological compartments, influencing cellular uptake/exocytosis, impacting cellular localization and up- or down-regulating immune system activity (examples are discussed below). The protein corona may also insulate the reactive surface of the nanoparticle and diminish chemical reactivity. For example, the redox activity of single, walled, carbon, nanotubes was suppressed after bovine serum albumin was adsorbed to the particle surface and formed thick and dense layers around the carbon nanotubes [60]. If the net reactivity of the NP is pro-oxidant, the protein corona may decrease cytotoxicity, but it may also suppress antioxidant activity [59].

Beyond the effects of protein adsorption on NP activity, Horie et al. [78] examined the effects of protein adsorption on metal oxide NPs (NiO, ZnO, TiO_2_, CeO_2_, SiO_2_, and Fe_2_O_3_,) on cell viability. The adsorption of the components of the culture media onto metal oxide NPs can induce a starvation state in a dose-dependent manner in cells-independent of direct cellular effects of the nanomaterial. The secondary effects of serum depletion on viability were impacted by the size of nanoparticles, the elemental composition of the NP, and the types of proteins adsorbed to the particles. Hence, examining the effects of potential contributions of serum depletion of media constituents is critical when interpreting data arising from in vitro experiments.

The ‘rules’ that dictate the process and nature of protein adsorption are not fully understood. Physical-chemical attributes of nanoparticles that influence generation of the protein corona include elemental composition, particle size, charge, hydrophobicity/hydrophilicity, and the nature of surface stabilizers used during NP synthesis. These factors can each independently influence protein accretion, making it difficult to identify unifying principles for all materials that might predict patterns of biochemical activity and leaving investigators with the daunting task of evaluating the contribution of these factors to protein-nanoparticle-cell interactions for each nanomaterial on a case-by-case basis. The behavior of pristine nanoparticles in biomolecule-free media, cannot, in most instances, be used to guide biological experimentation, and even cultured cells cannot be maintained successfully for extended periods of time in serum free (i.e., protein free) conditions. Hence, little or no useful guidance about bioreactivity in vivo can be gained for studies in protein-free conditions.

### 3.1. Remnants of the Protein Corona Persists Intracellularly

Using 50 nm polystyrene nanoparticles in A549 cells (adenocarcinoma human alveolar basal epithelial cells), Bertoli et al. [79] showed that while the initial stages of plasma interaction and endocytosis were impacted by the complement of proteins present on the surface of the nanomaterial, these proteins were not completely degraded from the NP surface even when the NP arrived in lysosomes. While a large fraction of existing proteins on the surface of the NP may be degraded within lysosomes, some protein fragments remain on the particle for extended periods of time both within the lysosome and the cytosol, leaving traces of the history of environments through which the particle moved, like the underwriting on a palimpsest.

The adsorption of intracellular proteins may have an even greater biological impact than adsorbed extracellular proteins. Recently, Qin et al. [80] used 30 nm Au NPs to study the intracellular protein corona in immortalized Caco-2 cells. Forty, intracellular proteins were adsorbed to the surface of the gold NPs. In addition, six extracellular proteins were detected within the intracellular protein corona, most of them associated with the extracellular matrix. While accretion of extracellular proteins may dictate route of initial cell entry or contribute to extracellular cell signaling, the secondary evolution of the intracellular protein corona may depend on residual extracellular protein effects even as intracellular proteins come to dominate particle trafficking or cell signaling.

### 3.2. Species-Specific Differences in Protein Adsorption

**Figure 3:**
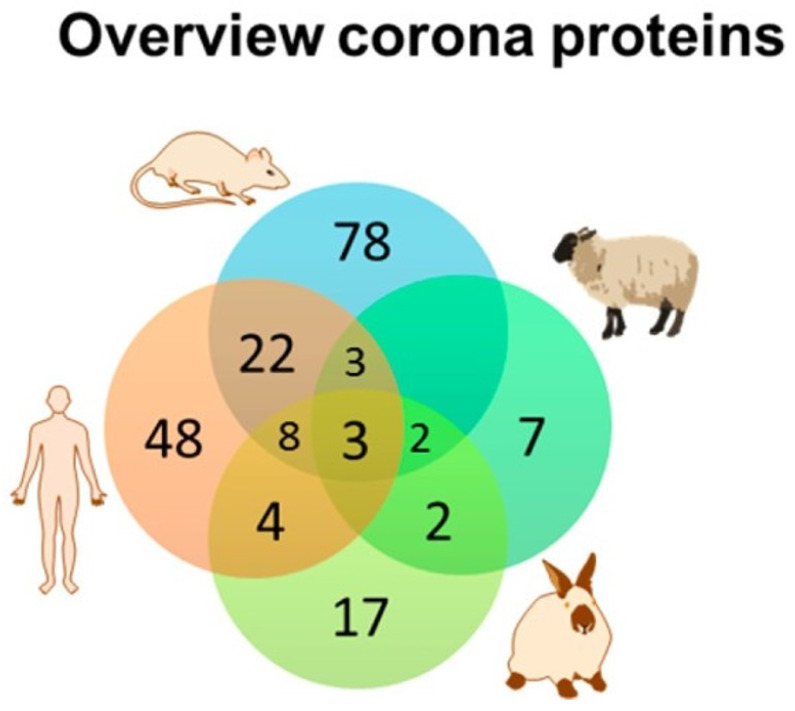
The similarity and diversity of the composition of the protein corona around polystyrene nanoparticles in four species are shown above, The Venn diagram indicates that few common proteins were identified among the different species studied even though identical NPs were studied in all four species. A similar diversity of corona proteins was identified when a dextran-coated magnetite NP was studied. Adapted from Muller et al. [81] with permission.

After incubation of two types of PEGylated lipid NPs in mouse or human plasma, more than 300 proteins were identified in the corona; however, only 34 and 28% of all the identified proteins were common on the NPs between mouse and human coronas; even among the 25 most-abundant corona proteins identified, only half of proteins were similar between mouse and human proteins. The mouse plasma corona was more enriched in apolipoprotein and less enriched in immune activating-opsonins compared with the human plasma corona [62]. In a similar study using plasma from rat, rabbit, sheep and human, the stability (resistance to aggregation) of two nanoparticles with multiple different surface functional groups, the composition of proteins adsorbed to the NPs were divergent and strongly dependent on the plasma source of the adsorbed proteins (Figure 3) [81]. The differences in the protein corona arising from incubation in the various plasma sources were much greater than the effect of surface functionalization of the NP. The results of these studies imply that there is limited transferability of results from animal to human studies and raise questions about the apparent capacity of studies in animal cells and tissues to predict behavior of NPs in humans [82].

### 3.3. In Vitro and In Vivo Differences in Protein Adsorption

In a study of the protein corona generated under in vitro and in vivo conditions in CD-1 mice, the protein corona formed on three liposomes (one bare-no functionalization, one PEGylated, and one decorated with targeted IgG antibodies) was compared after in vitro incubation in plasma from CD-1 mice or after brief (10 min) injection into the circulation [83]. Although the total amount of liposome-associated proteins did not significantly vary between in vitro and in vivo conditions, the composition of the protein corona was more complex after in vivo injection, and in both cases, the protein corona reduced receptor binding and cellular internalization. While this work has yet to be performed with metal oxides, these findings indicate that even within a species, the system studied matters; in vitro plasma incubation is a poor predictor of in vivo behavior.

### 3.4. Factors That Influence Protein Binding to Nanoparticles

#### 3.4.1. Surface Area/Size

With higher surface area: volume ratios and greater radii of curvatures, smaller particles tend to bind more proteins than larger particle with smaller surface area: volume ratios due to reduced steric hindrance except at very small sizes (1–2 nm) where the radii of curvature are too extreme for stable binding [61,84]. There may be a size limit below which a complete protein corona cannot fully develop due to geometric constraints. The protein corona of 2–5 nm gold NPs formed stratified protein layers (as opposed to an admixture of proteins) comprised of plasma proteins bound around the NPs [85]. The inner, hard corona also evolved more quickly around smaller Au particles (3.5–150 nm) compared to larger ones, and the protein corona was thinner surrounding smaller particles [86]. Gold nanoparticles less than 10 nm ride like a cargo on proteins rather than acting as a carrier for the protein. In contrast, Au nanoparticles greater than 10 nm transition to serve as protein carriers in which the proteins decorate the particles rather than the converse [87]. The behavior of the nanoparticle may differ depending on whether the nanoparticle was a cargo or a carrier. Small particles (4 nm, citrate-capped Au NPs) bound to a test protein did not alter the ligand binding of the protein to its cellular receptor, suggesting that the nanoparticle binding did not affect the conformation of the protein, and the proteins, rather than the nanoparticle, likely dictated the cellular fate of the NP [87]). Thus, particle size plays a critical role in both the kinetics and the thickness of the protein corona [78].

#### 3.4.2. Shape

Protein adsorption of albumin to spherical gold nanoparticles (50–70 nm) was three times higher than adsorption to branched gold NPs of similar size [88]. When zinc oxide pyramids, plates, and spheres were compared, different protein binding was observed depending on the nanoparticle shape [89]. Thus, the shape of the NPs and interactions with proteins based on NP shape can significantly impact the blood circulation time, cellular internalization, bio-distribution, endocytosis by immune cells, and residence time within cells [90].

#### 3.4.3. Surface Charge

Nanoparticles with charged surfaces tend to adsorb more proteins than those with neutral surface charges [91,92]. For example, only 47.2% of the serum protein corona was found common on cationic amine-functionalized Au NPs compared to anionic carboxy-functionalized Au NPs, and 65.9% of the serum protein corona formed around trimethylammonium-functionalized Au NPs was common to amine-functionalized Au NPs in the size range of 15–60 nm [93]. Proteins with a negative charge (pI < 7) were preferentially bound by negatively charged SiO2 NPs, studied at physiological pH (7.3), irrespective of their relative plasma abundance, while proteins with pI > 7 were less enriched [94]. Regardless of initial surface charge, all nanoparticles seem to develop a negative surface charge in biological solutions due to the accumulation of proteins to the surface of the material.

#### 3.4.4. Elemental Composition:

The elemental composition of the NP also affects the proteins bound to different NPs. Deng et al. [95] compared protein absorption among three different metal oxides (Si, Ti, Zn; 7–30 nm, zeta ~−25 mV). Despite these particles having similar surface charges in a buffer solution, each nanoparticle rapidly bound different plasma proteins, suggesting an important role for the elemental composition of the core. Only ~37% of the serum proteins within the corona formed around silver NPs (Ag NPs) were shared by gold NPs (Au NPs) of similar size and surface charge, even though the NPs were modified with the same surface ligand, demonstrating that the core material exerts significant influence on the composition of the protein corona [61].

#### 3.4.5. Hydrophobicity/Hydrophilicity

The hydrophobicity of NPs also modifies the proteins in the corona, and the effects of hydrophobicity or hydrophilicity are independent of surface charge [96,97,98,99]. In a recent study, two ligands were used to change the hydrophilicity of 7–8 nm Au NPs along a spectrum: hydrophilic ligand A was created with a tri-ethylene glycol, and hydrophobic ligand B was created with undecane-coated NPs. Nanoparticles with more hydrophobic surfaces adsorbed more proteins; small and negatively charged proteins were preferably adsorbed to NPs with hydrophobic surface properties. Hydrophobic surfaces adsorbed about twofold more proteins than hydrophilic surfaces [99], and the densely packed adsorbed proteins appeared to restrict exchange with other free proteins in the solution simply due to reduced mobility. Last, the protein exchange rate was higher for hydrophilic NPs compared to hydrophobic NPs, likely reflecting the generally higher hydrophilicity of proteins, which would have been excluded from the hydrophobic environment around such NPs.

#### 3.4.6. Composition of the Diluent

Strojan et al. [100] examined the protein corona generated on Si NPs and polyacrylic acid-coated cobalt ferrite NPs in various solutions (distilled water, NaCl, PBS, RPMI media). Protein was added to the diluents to generate equivalent 10% fetal bovine serum concentrations, and mass spectroscopy was used to identify proteins associated with the hard corona after SDS-PAGE electrophoresis to separate of the proteins. Even within a particular type of nanoparticle, the composition of the protein corona and the hydrodynamic diameter differed depending on the diluent. While the types of proteins comprising the corona were roughly similar between diluents and types of particles, the relative abundance of the proteins varied both by particle type and diluent.

#### 3.4.7. Other Factors—Fluid Kinetics

Shear forces of fluid tended to increase the negative charge of the protein corona of circulating PEGylated liposomes [101]. More weakly bound proteins will likely not associate with particles that are exposed to higher shear forces. The in vivo composition of the corona will not, therefore, be the same as the in vitro condition simply based on the effects of fluid shear forces. Static in vitro models poorly predict the protein corona composition in vivo, and in vitro models are currently being developed that include dynamic fluid exchange to try to mimic the effect of shear on the protein corona [102].

## 4. Mechanisms of Cellular Uptake and Trafficking of Nanoparticles

**Figure 4:**
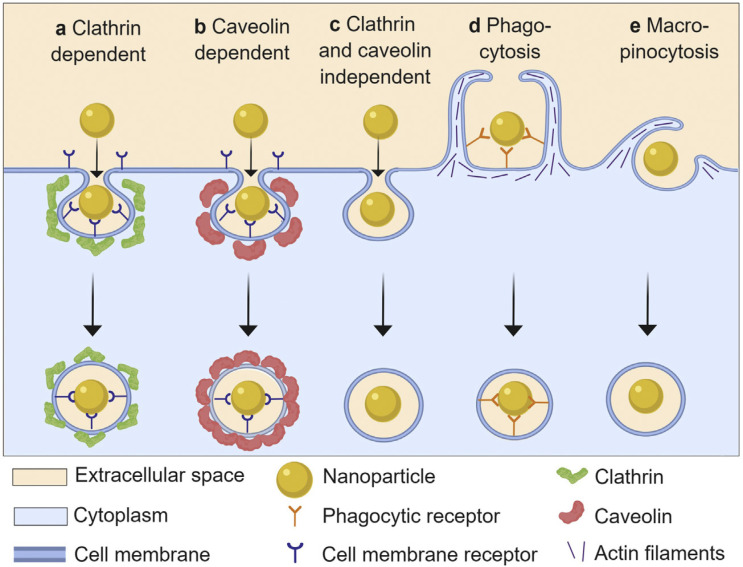
Schematic presentation of different endocytic pathways used by nanoparticles. The different uptake mechanisms dispose of nanoparticles intracellularly through different processes, and so the uptake mechanism has an important effect on intracellular activity and durability of metal oxide NP activity. Taken from Donahue et al. [106] with permission.

Nanoparticles enter cells by endocytosis, which can be broadly categorized into five mechanisms: clathrin-mediated endocytosis, caveolin-mediated endocytosis, clathrin/caveolae-independent endocytosis, pinocytosis and phagocytosis. Some authors group the first four mechanisms as subtypes of pinocytosis (Figure 4). Compared to phagocytosis, which is a primary function of ‘professional phagocytes’ (e.g., macrophages), pinocytotic (macro-micropinocytosis) mechanisms occur in virtually all cell types. The reader is encouraged to explore several excellent reviews on this topic [103,104,105]. In addition to these organized processes, some NPs may gain access to the intracellular space through direct membrane disruption or simple diffusion.

### 4.1. Clathrin Receptor-Mediated Endocytosis (CRME)

During CRME, vesicles with diameters of 100–150 nm are formed, engulfing extracellular fluid proportional to the available internal volume of the vesicle formed. Clathrin receptor-mediated endocytosis is the predominant mechanism of cellular uptake of nutrients, cholesterol, hormones, neurotransmitters, and iron. Extracellular molecules bind clathrin-receptor rich regions comprising ~0.5–2% of the plasma membrane [107], and after the molecules bind to clathrin-receptor coated pits, the ligand-receptor complex is engulfed by invagination of the cell membrane and formation of intracellular clathrin-coated vesicles. The cargo within these vesicles is shuttled through the endosomal system and terminates in acidic lysosomes.

### 4.2. Caveolin-Mediated Endocytosis

Caveolae are formed by assembly of caveolins, integral membrane proteins that bind directly to membrane cholesterol. In caveolin-dependent endocytosis, 50–80 nm, flask-shaped caveolae are formed on the plasma membrane [105]. Molecules internalized via caveolin-dependent endocytosis are susceptible to escape from degradation by lysosomes leading to cytosolic distribution. The caveolar pathway is responsible for the endocytosis of ligands such as albumin [108], autocrine motility factor [109], tetanus toxin [110], cholera toxin [111], and viruses.

### 4.3. Other Endocytic Pathways

The clathrin/caveolin independent pathway also contributes to cell entry of metal oxide NPs. The, clathrin/caveolin independent pathway is important in the uptake of cellular fluids, hormones, and folic acid, and folic acid is of particular interest given its role in targeting nanomaterials to certain forms of solid tumors [112,113]. Macropinocytosis and micropinocytosis play minor roles in the cellular trafficking of metal oxides in most studies, and consequently they will not be considered further in this review.

### 4.4. Phagocytosis

Phagocytosis is the main uptake process for particles larger than 500 nm. Phagocytosis is carried out primarily by macrophages, which identify and remove foreign organisms and remove cellular detritus. Immunoglobulins and complement proteins act as opsonins, which ‘tag’ foreign materials, and macrophages recognize the opsonized proteins, specific surface moieties, and other surface markers. Once opsonized, NPs interact with surface receptors on macrophages, the particles are internalized by the cell and degraded. The types of surface proteins bound to NPs contributed significantly to NP clearance by ‘professional’ phagocytes (like macrophages), and opsonization primed NPs for reticular endothelial system recognition and clearance [114].

Several macrophage receptors and opsins have been implicated in the sequestration of nanoparticles including Toll-like receptors, mannose/lectin receptors [115,116,117], immunoglobulins, and members of the complement cascade, in which the regulation of C3 activity may be central in activating the complement cascade [118]. PEGylation can decrease the binding of these proteins and reduce, but not eliminate, phagocytic clearance of NPs by the reticular endothelial system [119,120]. Having more control over the factors regulating active clearance of nanomaterials is important in optimizing dosing, reducing off target effects, and dampening alterations in immune system function [121,122,123].

### 4.5. Membrane Translocation

Nanoparticles may disrupt the cell plasma membrane by interacting with lipid bilayer molecules to allow direct transport of nanomaterials into the cytoplasm independent of endocytic pathways. This process avoids endosomal sequestration and the typical transport mechanisms used to gain access to the cytoplasm [124,125]. Diverse types of surface modifications can induce membrane translocation of metal oxides including cell penetrating peptides. Cell penetrating peptides, including penetratin, Tat, sC18, VP22, and the poly-arginines, have been used successfully to deliver a variety of proteins, polypeptides, nucleic acids organic/inorganic particles into the cell [126]. Last, some neutral zwitterions seem to enter cells by passive diffusion, whereas cationic and anionic NPs were taken up by endocytic processes [127].

### 4.6. Physical Attributes of NPs and Cellular Uptake

The protein corona—determined by the physical and chemical characteristics of each NP discussed above—is the surface that each NP presents to biological membranes, and the protein corona will determine how NPs enter cells. Hence, NP shape, size, surface charge, elemental composition and hydrophilicity will steer each NP to one endocytic process or the other [128]. There are, however, few easily predicted and consistent rules about cellular uptake of metal oxide NPs.

Particles between 10–50 nm exhibit the highest rates of entry in many cell types whereas large particles (~100 nm) and very small particles (1–2 nm) are taken up less avidly. The reduction in uptake of very large particles is attributed to agglomeration into amorphous particles that are too large to access classical endocytic pathways. Very small particles are also assimilated into cells less effectively than larger particles, although the reasons for this reduced accumulation are not known. Smaller metal oxides nanoparticles (<10 nm) tend to enter and exit the cell more rapidly than larger NPs [88], and the concentration of smaller NPs may be less at any instant even though the entry and exit of small NPs are more rapid. In nanogold particles, the protein corona significantly decreases cellular uptake efficiency in a size-dependent manner in in vitro studies. The accumulation of larger particles (~50 nm) was reduced by the presence of a protein corona whereas, the uptake of small particles (5 nm) was unaffected by their interactions with proteins [129]. The majority of blood proteins, albumin, immunoglobins, ApoE, etc. are large (100s of kD, 1000s of amino acid residues), so it is likely that smaller nanoparticles decorate these larger proteins and tissue targeting is dependent on the protein. It is possible that nanoparticles may change from cargo to carrier, and in the transition, the targeting and activity of the protein may be dictated by the NP, especially once the NP enters the intracellular space where intracellular proteins in eukaryotic systems tend to be small (196 to 1157 amino acids) [130]. The biological impact of the corona on cellular accumulation is further complicated in that the nature or amount of protein associated with the material, and any change in protein structure associated with nano-bio interactions may modify the ‘usual’ endocytic pathway for a particular protein. Last, the cell type studied greatly impacts the endocytosis of nanomaterials: the protein corona of larger gold NPs (50 nm) decreased uptake in phagocytic cells to a much greater extent than non-phagocytic cells [131].

Surface charge of the NP matters as well: positively charged nanoparticles exhibited much higher rates of endocytosis and retention than negatively or neutrally charged nanoparticles. Often, longer retention was associated with agglomeration of the particles within the cell, which makes active elimination more difficult [88].

Shape can also affect cellular uptake in immortalized cells [132], and shape may influence the rate of clearance from the circulation as well as nanomaterial uptake by immune cells [133,134,135]. Gold nanotriangles exhibited the greatest cellular uptake by RAW264.7 (immortalized macrophages), followed by gold nanorods and gold nanostars [136]. All three shapes entered cells through clathrin-receptor mediated endocytosis. In addition, gold nanorods were taken up by caveolae/lipid raft-mediated endocytosis, and gold nanotriangles uptake was strongly associated with cytoskeletal rearrangement by the clathrin receptor-mediated pathway and caveolae/lipid raft-mediated endocytosis, as well as the dynamin pathway associated with phagocytosis and pinocytosis. Spherical NPs, such as gold or PEGylated NPs, have higher uptake rates than other shapes [137]. However, other authors propose that non-spherical NPs accumulate in cells to a greater extent than their spherical counterparts [138]. The reasons for these discrepancies are not clear, but are likely associated with the cell types screened, differences in surface modifications (both synthetic additions to the NP and the number and types or proteins adsorbed to the surface), or particle deformability.

Like other chemical attributes, the hydrophilicity or hydrophobicity may affect cellular uptake. For example, gold NPs coated by hydrophilic materials demonstrated virtually no passive transport across artificial phospholipid membranes [139] or HeLa cells [140]. Moreover, nanoparticle elasticity and hydrophobicity affected the translocation across lipid bilayer in membranes using molecular dynamics simulations [141]. Hydrophobicity and deformation had interactive effects on transcytosis of NPs across simulated lipid membranes: hydrophilic NP penetrance across lipid bilayers was enhanced by increasing the NP stiffness, whereas the movement of hydrophobic NPs diminished with increasing stiffness [141]. Nanoparticle rigidity seems to increase endocytosis in comparison to soft NPs. Rigid NPs are more likely to be taken up by clathrin-receptor mediated endocytosis while more flexible NPs are endocytosed by micropinocytosis [142]. Micropinocytosis engulfs macromolecules and particles less than 0.2 μm in diameter. Theoretical studies defining the role of particle elasticity in drug delivery focus on the adhesive wrapping of a deformable particle by a lipid (cell-like) membrane in 2D and 3D models [143,144]. The simulations demonstrated that the soft NPs deformed during cell membrane interactions and were less likely to be endocytosed, whereas rigid NPs enter cells more readily. To date, the extent to which these features influence bioaccumulation of metal oxides NPs has not been determined.

Last, surface modifications of the NP may change the endocytic pathway through which a NP enters the cell. As shown in Table 1, the uptake of gold NPs depended on the cell line studied, the nature of the surface modification of the NP (and presumably the nature of the proteins bound to the NP-though this was not studied), and the NP size all seemed to lead to different endocytic or non-endocytic pathways into the cell [106]). Note the diversity of processes involved based on rather subtle changes in NP characteristics.

### 4.7. Endocytosis, Cellular Localization and Mimetic Activity of Metal Oxides NPs

The physical-chemical characteristics of each metal oxide NP and valence state dictate the redox effects of a given particle, but cellular uptake and localization can significantly affect surface reactivity, protein adsorption and intracellular trafficking and in doing so, bias the redox reactivity in one direction or another (i.e., anti- or pro-oxidant) [145]. A number of studies have examined nanoceria localization with different proteins and organelles in a range of different cell lines [146,147,148,149,150]. The cellular accumulation of nanoceria in transformed human ovarian and colon cell lines occurred via a multiple endocytic pathways including clathrin-receptor mediated uptake and caveolae. The CeOx NPs (7–94 nm) were localized predominantly in the cytoplasm and, to a lesser extent, in lysosomes. The intracellular trafficking varied as a function of particle size, treatment time, surface modification and cell type. Approximately one-third of internalized FITC-nanoceria colocalized with lysosomes. When nanoceria was present in the cytoplasm of human endothelial cells, the CeOx NPs exhibited SOD and catalase-like behavior [149]; whereas, when the nanoceria colocalized with the cytoplasm and lysosomes, it behaved like an oxidase. The change in preferred enzyme-mimetic activity likely reflects the effect of pH on the ROS-scavenging properties of nanoceria in different cellular locations. Based on the predominantly cytosolic distribution, CeOx NPs are probably trafficked via caveolin mediated endocytosis and/or CRME processes with endosomal escape. While the net effects of cellular localization can drive pro- or antioxidant behavior of CeOx NPs, the findings of this study showed that the CeOx NPs retained a net antioxidant effect (reduced cellular ROS levels), though this in not uniformly found [34].

Using the dynamin inhibitor, dynasore, Ding et al. [151] studied uptake of gold NPs of different sizes and shapes in three types of cells: SMCC-7721 (human hepatocellular carcinoma), GES-1(human gastric epithelium), and 4T1 (murine breast cancer). Uptake of the Au NPs was dramatically reduced in immortalized cell lines after treatment with dynasore. The NPs were endocytosed by multiple processes–phagocytosis, pinocytosis, and clathrin- and caveolin-dependent processes–depending on the size, shape, agglomeration, and surface properties of each NP. Clathrin- and caveolin-dependent processes are inhibited by dynasore, and chlorpromazine inhibits clathrin-dependent uptake. Treatment with chlorpromazine reduced NP uptake, though less than dynasore. This finding suggests that CRME and caveolin-mediated endocytosis, both of which are dynamin dependent, predominate in the cellular trafficking of Au nanoparticles in immortalized cells. Last, the presence of a protein corona stabilized the NPs, prevented aggregation, and reduced cellular uptake. This single study revealed both the variety of endocytic processes internalizing metal oxide NPs and the complexity among the physico-chemical attributes and protein corona of each NP and endocytic mechanism.

## 5. Cellular Removal of Nanoparticles-Exocytosis

While the endocytic processes and organelle localization of metal oxide NPs have been studied using many different nanomaterials, the mechanisms underlying the trafficking of nanoparticles out of cells have not. Exocytosis is the processes by which cytoplasmic vesicles and granules fuse with the plasma membrane, either spontaneously (constitutive exocytosis) or in response to cell stimulation (regulated exocytosis). Constitutive exocytosis occurs by formation of membranous secretory vesicles within the cell, in which the cargo is packaged with proteins and then continually released to the extracellular space. Constitutively released proteins are synthesized in the endoplasmic reticulum and transported through the Golgi complex to be packaged at the trans-Golgi network into the secretory vesicles. Regulated exocytosis or regulated secretion is an essential process that occurs in specialized secretory cells in response to an extracellular stimulus. Regulated exocytosis involves receptor-mediated stimulation of granule mobilization and fusion with the plasma membrane. In this case, cargo proteins are synthesized when needed by the cell and packaged into granules for subsequent release. Regulated exocytosis occurs in hematopoietic, neuronal, endocrine and exocrine cells [152]. Interactions among the elemental composition, size, shape, surface characteristics, and cell type fully as complex as interactions affecting uptake also affect NP exocytosis [153]. Wang et al. [154] measured the intracellular uptake and excretion of CuO NPs in A549 cells and found that a portion of NPs, which were located in mitochondria and the nucleus, could not be excreted by the cells. Nanoparticles that leave the endocytic vesicles or lysosomes and translocate into the cytoplasm are retained for longer periods of time and removed by exocytosis less rapidly [155]. In a typical exocytotic process, the NPs are initially trapped in lysosomes before transportation to the cell membrane for excretion. Often NPs that are trapped in lysosomes need to associate with a large number of proteins and receptors for eventual exocytosis. Thus, clusters of silica NPs in lysosomes were more easily removed by exocytosis by H1299 cells (model of small cell lung carcinoma) compared to single NPs in the cytoplasm [156]. Moreover, 4 to 22 nm size gold nanoparticles were degraded in vitro by lysosomes in fibroblasts, and degradation was faster for smaller particles. Furthermore, products of Au NP degradation increased cellular oxidative stress, and the gold recrystallized into biopersistent nanostructures that were not readily cleared from the cells [157]. Roughly shaped, nanodiamonds seemed to disrupt endosomal membranes in which they were encapsulated initially and broke into the cytoplasm where they were seldom subject to exocytosis [158]). The cellular translocation and excretion of nanodiamonds were completely different from those described for spherical Si NPs, which were localize primarily to endosomes or lysosomes once inside the cells, seldom found in cytoplasm, and readily expelled by exocytosis. The authors concluded that morphological characteristics of the nanodiamonds (e.g., sharp corners versus rounded corners of conventional Si NPs) may have contributed to the lack of exocytosis of this more irregularly shaped material (Chu et al., 2011).

Ding et al. [151] screened the uptake and release of 5 different Au NPs preparations that varied in size and shape. They used the microtubule inhibitor nocodazole in three, immortalized cell types (GES-1, 4T1, 7721) to inhibit microtubule formation and disrupt fusion of the lysosome with the plasma membrane [159]. Nocodazole pretreatment significantly suppressed exocytosis of all five types of Au NPs indicating that the bulk of Au NPs were lost or removed from the cell through lysosomal fusion. Similarly, Strobel et al. [160] evaluated the exocytosis of CeOx NPs in immortalized human microvascular endothelial cells (HMEC-1) used nocodozole and Brefeldin A, which blocks the intracellular transport of secretory, lysosomal, and membrane proteins beyond the endoplasmic reticulum and causes the loss of Golgi membranes in most cell types. The effects of Brefeldin A are non-specific but impact endocytosis [161]. Using TEM and flow cytometry to study CeOx NP exocytosis, the transfer of CeOx NP in HMEC cells in distal Golgi compartments was reduced by Brefeldin A treatment, and the loss of intracellular CeOx NPs after Brefeldin A treatment was greater than after nocodozole treatment [160]. The involvement of the Golgi in nanoparticle exocytosis seems to be a conserved pathway that plays a role in cellular egress for other types of non-metallic, nanoparticles as well [162,163].

### Limitations of Immortalized Cell Lines

Many of the foregoing studies examined cellular localization and NP deposition only in transformed, immortalized cells. The magnitude and pathways of endocytosis (and possibly exocytosis) appear to vary between cell lines and native, non-transformed cells. Using 29 lung cancer cell lines and comparing them to normal, isogenic lung cell cultures, Elkin et al. [164] found significant differences in endocytic pathways depending on cell type (immortalized or normal) and the biological environments in which the cells were maintained (in vitro or in vivo). Many components of the endocytic machinery were mutated or had altered expression in cancers [164,165,166,167]. Significant differences in the expression of many endocytosis-related genes exist between immortalized cells and primary cultures [168], and signaling downstream of surface receptors involved in endocytosis can be dysregulated in cancer cells [169]. For example, identical NPs trigger different responses in different immortalized cell lines, casting doubt on the use of cell lines to emulate normal, physiological functions consistently [45,170,171,172]. Cell lines may be preferred over primary cell cultures because of their homogeneity and greater stability, which yields better experimental reproducibility as compared to primary cell cultures in which there is greater batch-to-batch variability, lack of tissue availability, limited number of cells yielded from each preparation, donor-specific variations, and a relatively short usable life of the cultures. However, immortalized cells differ in their genetics, proteomics, and function, and their use as comparative, surrogate systems should proceed only once similarities in cellular function with their non-immortalized counterparts have been verified. When responses to and effects of TiO2 NPs were examined in cancer or immortalized cell lines versus primary cell lines representing the same tissue and species (human primary bronchial epithelial cells compared to lung epithelial cell lines A549 and BEAS-2B), the responses to the titanium dioxide NPs were quite different among the different cells [173,174]. When six related neural cell types from immortalized or primary neural cells were exposed to iron NPs, cell morphology, acute toxicity, redox state, and intracellular calcium levels all varied widely among the closely related cell types [175]. The authors concluded that developing a set of standard cell types that reflect normal, healthy target tissues is required for the generation of meaningful toxicity profiles. Similarly, the biological effects of three different nanomaterials (Ti, Si and MWCNT nanomaterials) had variable biological effects (e.g., cell viability, ROS production, mitochondrial function) depending on the cell type studied (3T3 fibroblasts, RAW 264.7 macrophages, and telomerase-immortalized (hT) bronchiolar epithelial cells were compared) [176]. These studies emphasize the need for caution when using immortalized cells as model systems to predict the translational efficacy of metal oxide NPs. Determining meaningful endpoints that reflect physiological outcomes or processes of interest in vivo remains a challenge, and few investigators validate the experimentally observed phenotype against primary cell types and therefore fail to produce biological outcomes representative of in vivo responses [177].

The mechanisms of cellular uptake and the activity of particular transport processes vary dramatically among the cell types. Thus far, there has been a scatter-shot approach to studying cellular trafficking of nanomaterials based on the assumption that these processes are similarly active among all cells regardless of origin, culture history and function. This is probably a poor assumption to make [178]. Even within a single cell type, different pathways of endocytosis are utilized depending on whether the cells are dividing, quiescent, fully differentiated, or senescent. During cell division, endocytosis is largely dedicated to the recycling of receptors and caveolae. In terminal, non-dividing cells, clathrin-mediated endocytosis operates in concert with endocytic pathways, but the balance among different transport routes of entry and activity can vary widely depending on cell types. For better or worse, the preponderance of studies examining cellular trafficking of nanomaterials have used cell cultures derived from cancerous tissue. In our opinion, differentiated cells should not be compared with stem cells and neither of these cell types should be compared with professional phagocytes (i.e., immune cells) when characterizing the uptake of metal oxide nanoparticles. A standardized approach in cells with transport mechanisms that have not been modified by immortalization would be beneficial when studying cellular trafficking of metal oxide NPs; it will be difficult to compare routes and extent of cell entry between studies without some consistency among the methods used. Even with standardization, variance among cell types will need to be evaluated.

## 6. Finding a Path

Given the complexity of biological systems, it would be useful to bring some order to the diverse and chaotic array of NP characteristics and test systems. However, it is difficult to understand how a ‘model’ system can be developed since virtually every aspect of the NP, its basic chemistry and physics, the protein corona around the NP, the transport mechanism(s) and target cell(s), organelles within the cell and intracellular modifications of the protein corona modulate or even completely invert the expected activity of metal oxide NPs. The relative impact of each of the many factors influencing NP reactivity has not been determined and doing so would be difficult given the large number of variables involved.

Employing machine learning may prove useful in parsing the independent effects of the diverse array of factors that influence the reactivity, distribution, and bioaccumulation of metal oxide NPs. Machine learning approaches for both dichotomous and continuous variables have emerged as the analytical tool of choice to deal with such large multidimensional solution spaces. Machine learning has the advantage that it can be used in an unsupervised setting—one does not have to have a priori prediction rules to train algorithms and develop insights from these methods. Machine learning has been applied in several biological and chemical settings with success [179,180]. Compared to other traditional linear regression approaches with poor prediction performance (R2 values less than 0.40), machine learning can achieve good prediction performance especially when meta-analysis is incorporated into the model. Using this approach, machine learning models predicted the content of the protein corona on a given nanoparticle with an R2 of 0.75 [181]. In addition, the model successfully predicted the biological outcomes of the corona. Gainza et al. [182] had similar success in developing algorithms (termed Molecular Surface Interaction Fingerprinting; MaSIF) based on a geometric deep learning method to identify protein fingerprints that are important for specific biomolecular interactions. They demonstrated the capacity of the MaSIF algorithm to predict protein pocket-ligand interactions, the sites of protein-protein interactions. and likely protein-protein partners based on the presence of fingerprints in the protein surface. While this field is still in its infancy and approaches such as MaSIF have yet to be applied to interactions at the bio-nano interface, these approaches may prove to be powerful. As researchers begin to expand and improve the data sets associated with different nanomaterials, machine learning approaches will become more accurate and useful in designing biocompatible nanomaterials with predictable biological and therapeutic actions [181].

### An Instructive Example or a Cautionary Tale

Over the past four years, there have been 98 new nanoparticle formulations cleared by the FDA or EMA [183]. Of these, only thirteen drugs were metal oxides. The majority of approved metal oxide NPs are used to treat iron deficiency (eight nano iron formulations), two are used for cancer imaging (Fe NPs), and three are used to treat localized soft tissue cancers using electron or thermal ablation (1 Si/Au NP and 2 Halfnium NP formulations). The mechanisms of action underlying these compounds are straightforward. The high surface area and rapid dissolution of the Fe NP enhances free Fe formation, which increases the cellular incorporation of iron and ameliorates iron deficiency. In the case of tracking and ablation, the utility of the FeNP relies more on the physics, magnetic induction, of the nanoparticle than on its chemistry. There have been multiple off-label uses of Fe NPs including tracking stem cells and phagocytes and more recently as a potential antiviral for COVID-19 infection [184,185,186]. In terms of use of metal oxides in oncological settings, deposition of metals in and around cancer cells reflects local, increased vascular permeability in tumors and enhanced uptake of metal oxides by cancerous cells. While the use of metal oxides as theranostics may be possible, the current, medically important compounds harness only the rudimentary, physical characteristics of these nanomaterials.

It is instructive and ironic that the FDA-cleared NPs demonstrate little of the complex chemistry that make metal oxide NPs such attractive drug candidates; the FDA-cleared particles are coated with dextran or other substances to suppress all the complexity and interesting chemistry at the surface of metal oxide NPs. Thus, while the theoretical benefits of metal oxides in nanomedicine are enormous, the complexity of the interactions of these materials with biological substrates is daunting and has precluded approval of all but the most mundane metal oxide NPs.

## 7. Conclusions

The central theme of this review is the translational limitations of studies that describe the activity of metal oxide NPs in quite restricted and ‘non-biological’ settings and then proclaim the great biological potential for the NP in intact physiological systems. Reductionism has been a successful and fundamental approach identifying the mechanism of action in drug development, and many investigators use reduced systems to isolate the essential participants in any physiological process. While it is not a fault to use experimental paradigms that have proved successful in drug development in the past, it is a fault to persist with reduced preparations when it is clear that such systems limit the goal of developing therapeutic metal oxide NPs. Given the often unique interactions of nanomaterials and biomolecules for the vast majority of engineered, human-made materials, no single test in cell culture, a tissue or an organ can hope to predict the likely biological activities (and toxicities) of nanomaterials in the whole animal. Part of the ubiquitous reliance on in vitro tests originates in the desire to limit animal use in experimental research. The Environmental Protection Agency (EPA) indicated that they would like to decrease funding of studies in mammals by 30 percent by 2025, and completely eliminate funding for mammalian studies by 2035 (https://www.epa.gov/research/epa-new-approach-methods-efforts-reduce-use-animals-chemical-testing, accessed date 1 May 2021). While we applaud the goal of reducing, replacing, and refining use of animals in research, this approach will likely not be successful in nanoparticle drug development. Given the limitations of in vitro study design and interpretation, in vivo models will remain essential to bridge the large gap between whole animal and more reduced models. To harness the full potential of metal oxide NPs, investigators need to adopt a consistent combination of in vitro and in vivo models and analytical methods (like machine learning) to predict the behavior of metal oxide NPs in intact animals. When the predictive value of in vitro models relative to in vivo performance has been established, in vitro systems can be used to optimize particle delivery and activity at the desired target with some confidence that the findings will translate to in vivo settings. Until that future time, physiological approaches to predicting metal oxide NP behavior will remain an essential element in developing the unrealized potential of metal oxide NPs for use in humans.

## Figures and Tables

**Table 1:** Even for nominally similar nanoparticles, a wide array of uptake processes may be involved based on rather subtle changes in NP characteristics.

Major Uptake Pathway	Cell Line	Surface Modification	Core Particle Size (nm)
Caveolin dependent	HeLa	Cysteine-cyan 3	4.5
Caveolin dependent	HeLa	Cationic monolayer	2
Caveolin dependent /Lipid rafts	C166 (endothelial cancer cells)	Nucleic acids	10
Caveolin dependent/ Pinocytosis	A459 (lung cancer epithelial cells)	Poly (isobutylene-alt-maleic anhydride)	13
Clathrin dependent	MRC-5 (fibroblasts)	Fetal bovine serum	20
Clathrin/Caveolin independent	HUVEC (human umbilical vein endothelial cells)	citrate	80
Clathrin dependent	MCF 10 (non-tumorigenic epithelia cell line) Primary, mouse macrophages	Cationic monolayer	2
Clathrin/ Caveolin independent	HeLa	Cationic monolayer	2
Phagocytosis	Mouse primary macrophages	5-aminovaleric acid, L-Dopa, Melatonin, Serotonin-HCl	30–50
Direct translocation	Mouse dendritic cells	11-mercapto-1 undecanesuphonate and 1-octanethiol	4–5
Direct translocation	HCT-116 (human colon cancer cells)	Glutathione, glucose	5

Abbreviations: HeLa: human cervical cancer cells, C166: mouse endothelial cells, A549: adenocarcinoma human alveolar basal epithelial cells, MRC-5: human lung fibroblasts, HUVEC: human umbilical vein vascular endothelium cells, HCT-116: human colorectal carcinoma, L-DOPA: (S)-2-amino-3-(3,4-dihydroxyphenyl)propanoic acid, Melatonin: N-acetyl-5-methoxytryptamine, and Serotonin HCl: 5-hydroxytryptamine hydrochloride. Taken from Donahue et al. [106] with permission; please see original paper for references.
